# Left Paraduodenal Hernia Presenting as Closed Loop Jejunal Obstruction in a Young Female: An Enigmatic and Perilous Differential of Acute Abdomen

**DOI:** 10.1055/s-0041-1733989

**Published:** 2021-09-01

**Authors:** Deepak Rajput, Ankit Rai, Amit Gupta, Subramanian Chezhian, Shashank Kumar, Ashikesh Kundal

**Affiliations:** 1 Department of General Surgery, All India Institute of Medical Sciences Rishikesh, Uttarakhand, India

**Keywords:** closed-loop obstruction, small bowel obstruction, paraduodenal hernia, chronic pain abdomen, internal hernia

## Abstract

Internal hernia is a rare cause of intestinal obstruction, accounting for <2% of cases with paraduodenal type being the most common. An internal hernia, mostly acquired, develops due to protuberance of the intestine through a gap in the peritoneum or mesentery formed as a result of an antecedent abdominal operation such as gastric bypass or liver transplant, ischemic injury, peritonitis, or trauma. Paraduodenal hernias (PDHs) are congenital anomalies, secondary to a failed fusion of mesentery with parietal peritoneum along with rotational midgut errors, causing the evolution of potential space for herniation within the left paraduodenal fossa. Primary internal hernias can have a varied clinical presentation and cause significant mortality and morbidity if left untreated. We report the case of a 20-year-old female with chronic pain in abdomen and intestinal obstruction due to left PDH (LPDH). The prompt diagnosis led to timely exploration and reduction of entrapped jejunum, with prudent closure of the hiatus, while circumventing any injury to the adjacent mesenteric circulation. No postoperative ileus arose, and recovery was uneventful.

Although paraduodenal hernia (PDH) is rare in adults, it must be kept as a differential of any small bowel obstruction (SBO), particularly in patients with an antecedent history of abdominal trauma and absence of previous abdominal surgery and in patients with suspicion of biliary colic, gallstone disease, malignancy, bezoar, or significant weight loss.

## Case Report

A 20-year-old unmarried female, without any comorbidity, presented to the emergency with colicky-type upper abdominal pain radiating down the lower back for the last 2 years. She used to take nonsteroidal anti-inflammatory drugs (NSAIDs) to get relief from pain. For the last 1 week, the pain was continuous and associated with nausea and multiple episodes of nonprojectile bilious vomiting. She also had obstipation and decreased urine output over the previous 4 days. Abdominal examination revealed mild distension in the upper part with diminished bowel sounds.

Multiple air–fluid levels were detected on the left side of an abdominal roentgenogram; however, no free air was there. Laboratory workup revealed normal serum lipase (sent considering chronic pancreatitis as differential), hemoglobin: 0.97 g/L, total leucocyte count: 7.5 × 10 ^9^ /L, and serum potassium: 2.9 meq/L. Intravenous fluids and Potassium correction over the subsequent 48 hours led to the return of bowel function and improved urine output. Liver and renal function tests were within the normal range.

**Fig. 1:**
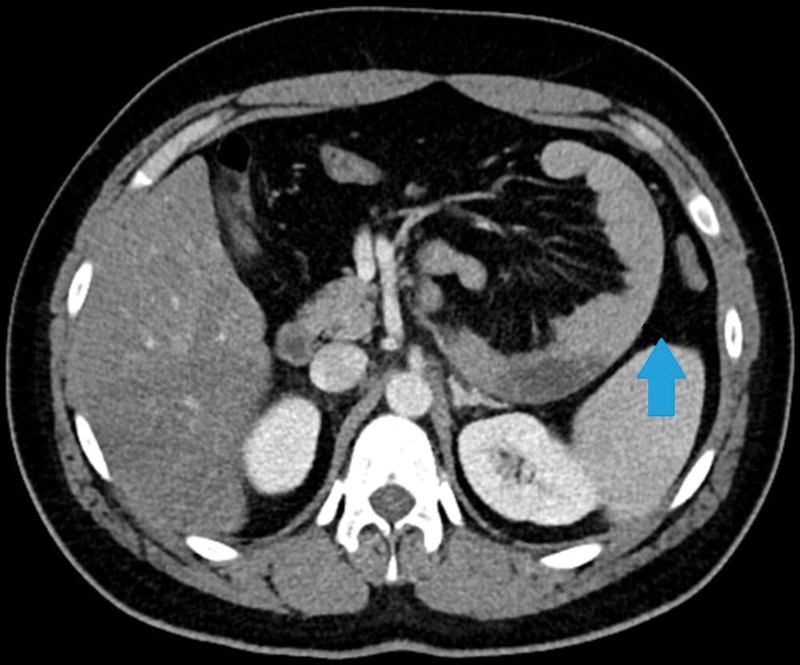
Axial CECT abdomen section showing abnormally located jejunum in left anterior pararenal space (blue arrow). CECT, contrast-enhanced computed tomography.

Ultrasound of abdomen did not reveal gallstone disease and upper gastrointestinal (GI) endoscopy was unremarkable. The patient tolerated liquids orally but postprandial pain persisted. She had stable vitals; hence, a contrast-enhanced computed tomography (CECT) scan of the abdomen was planned to keep proximal bowel obstruction as the provisional diagnosis. CECT scan of the abdomen demonstrated abnormally located jejunal loops in the left anterior pararenal space displacing the inferior mesenteric artery (IMA) anteriorly and convergence of mesenteric vessels in that region. Two transitional segments of narrowing were seen along the course of jejunoileal bowel loops closely approximated to each other on the left side of the abdomen. The intersegment bowel loop was dilated and arranged in **C** -shaped configuration, suggesting a closed-loop obstruction ( Fig. 1 ). The transverse colon was reaching up to the pelvis.

**Fig. 2:**
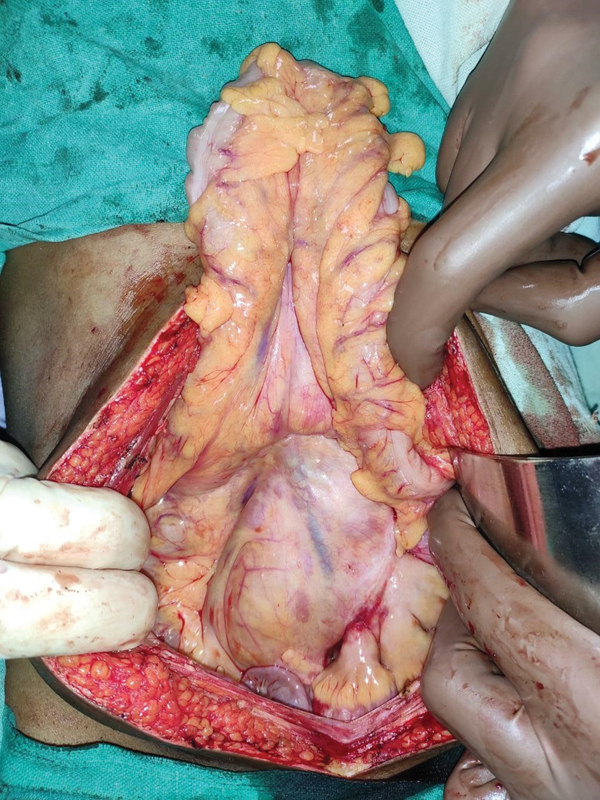
Exploratory laparotomy revealed a cluster of small bowel loops enclosed by the hernial sac in the retrocolic position with a redundant transverse colon above.

**Fig. 3:**
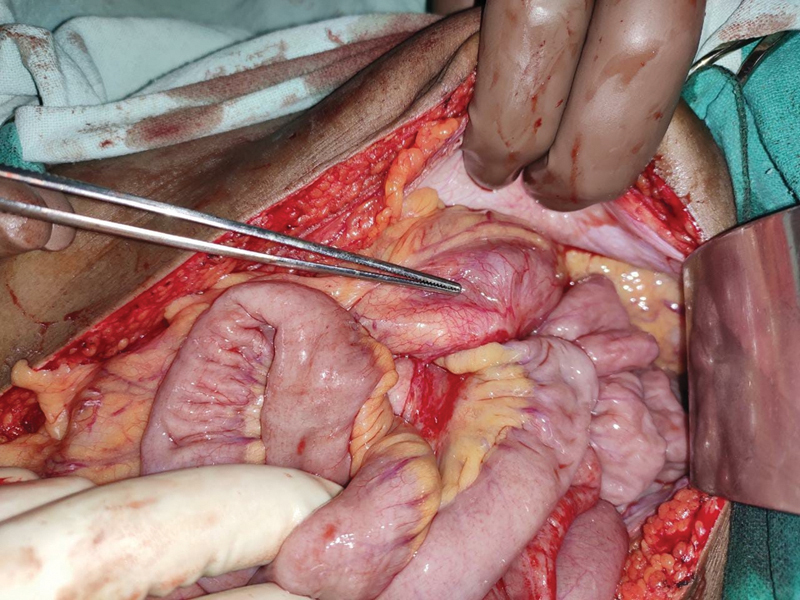
Postadhesiolysis proximal jejunum seen herniating into the mesenteric sac depicted by the tip of forceps.

**Fig. 4:**
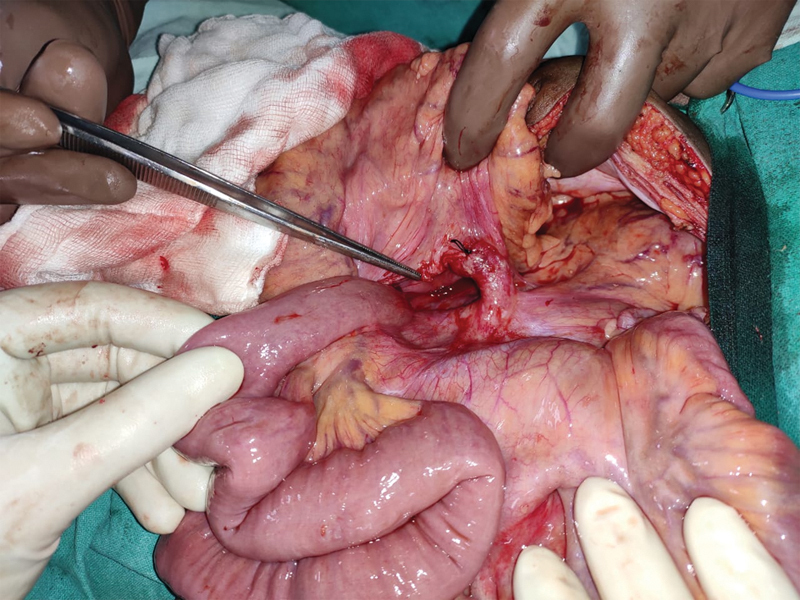
Tip of the forceps pointing toward Landzert's fossa and ascending left colic artery seen running over the ventral rim of the defect.

**Fig. 5:**
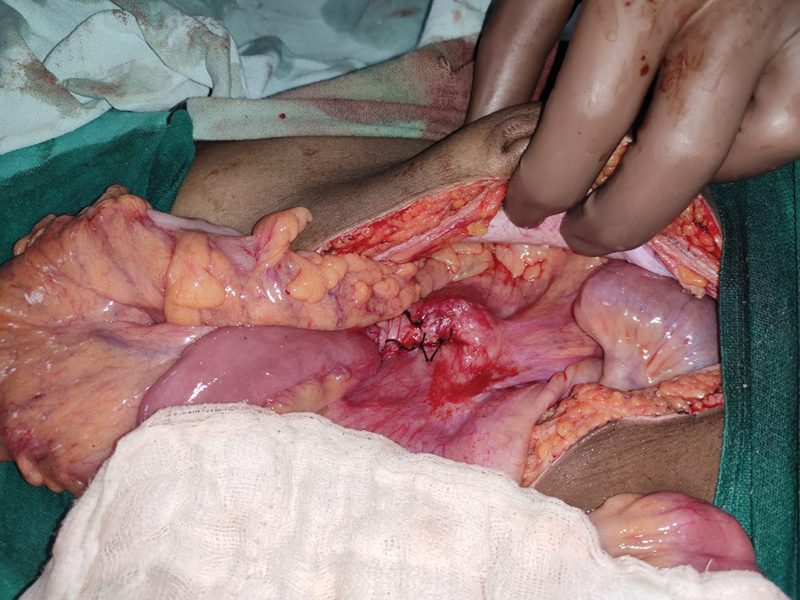
Obliteration of hernial orifice by approximation of mesocolon and base of the mesentery using interrupted silk sutures.

Because of the ongoing novel coronavirus disease 2019 (COVID-19) pandemic, the patient underwent open exploration via a vertical midline incision. Laparotomy revealed a congregation of small bowel loops enclosed within the hernial sac in a retrocolic position over the left paraduodenal area ( Fig. 2 ). Meticulous adhesiolysis demonstrated proximal jejunum herniating into the mesenteric sac ( Fig. 3 ). PDH neck was widened carefully by incising mesocolon through an avascular section, jejunum inspected for viability and reduced back into the peritoneal cavity. The ligament of Trietz was found in its usual anatomical location and ascending left colic artery was seen running over the ventral rim of the defect ( Fig. 4 ). The base of the mesentery and mesocolon was approximated and primary closure of the hernial orifice was done with interrupted nonabsorbable silk sutures, taking due care not to injure the adjacent inferior mesenteric vein ( Fig. 5 ). No complications ensued postoperatively, and the patient got discharged in satisfactory condition.

## Discussion

PDHs with an overall incidence of <1% are an infrequent cause of SBO, accounting for just 5.8% of cases. 1 They are the most common type of internal hernias, constituting nearly 53%, with the left and right subtypes accounting for 40 and 13%, respectively. 2 Incidence of left PDH (LPDH; Lanzert's hernia) is three times higher than of the right subtype (Waldayer's hernia). Lanzert's fossa is an area of mesenteric sac between the mesocolon and posterior abdominal wall where small bowel loops may herniate and get trapped. 3 It lies to the left of the fourth part of the duodenum and posterior to the inferior mesenteric vein and the ascending branch of the left colic artery.

PDH has an entirely ambiguous presentation with an obstruction risk of 50% and an overall mortality risk of 20% associated with left PDH. 4 Most common presentation of PDH is nonspecific pain in abdomen that may evolve into the partial or complete intestinal obstruction as was in our patient. PDH has been reported three times more common in males compared with females and the majority of cases present between the fourth to sixth decade of life with a mean age of 38.5 years. 5 An exhaustive literature search discovers cases of LPDH reported in female patients but the majority occurring in middle-to-old age. There are only occasional reports of PDH in young females. Satapara et al and Al Otaibi et al reported LPDH in young female patients. 6 7 Kozman and Fisher reported a case of LPDH postlaparoscopic appendectomy in a 15-year-old female patient. 8 We report a case of LPDH in an atypical patient profile, that is, young and female.

On X-ray of abdomen, congregated small bowel loops may be visualized in the left upper abdomen. Upper GI barium studies and CT are considered better for the evaluation of internal hernias. In typical CT images, PDH shows a cluster of dilated bowel segments with engorged and displaced mesenteric vessels at the hernial orifice. 9 Multislice CT (gold standard) offers high resolution and multiplanar images, thereby demonstrating characteristic radiological signs of hypoperfusion and intestinal ischaemia. 10

Surgical repair involves closure of the paraduodenal orifice after reduction of herniating contents. Occasionally reduction of engorged loops of the bowel may be difficult and can be accomplished by incising the mesocolon through an avascular section distal to the lower edge of the paraduodenal defect (done in our case), thereby preventing injury to mesocolic vessels. 11 Risk of incarceration and bowel ischemia mandates the repair of incidentally noted paraduodenal defects. 12 While the routine management of PDH involves open exploration, successful laparoscopic repair without any vascular compromise has also been reported. 13

## Conclusion

Internal hernias presenting as the acute abdomen is a relatively rare entity. Due to the nonspecific clinical picture, PDHs require a high index of suspicion and relevant imaging for establishing a correct diagnosis and early intervention. The patient profile in most of the published case reports on this entity is an elderly age group and male gender; however, the patient involved in the current case study is a young female who presented with hypokalemia. The laparoscopic approach can be considered in hemodynamically stable patients when expertise in advanced laparoscopic techniques is available.
